# Effects of Rapid Quenching on Grain Boundary Microstructure and Mechanical Properties of an Al-Mg-Si-Cu Alloy

**DOI:** 10.3390/ma16165609

**Published:** 2023-08-13

**Authors:** Qiao Yan, Yu Qiu, Mingjun Yang, Qiang Lu, Han Lin, Mingbo Yang, Kai Li, Yong Du

**Affiliations:** 1 State Key Laboratory of Powder Metallurgy, Central South University, Changsha 410083, China; 213311028@csu.edu.cn (Q.Y.); qwertyuiop20001213@163.com (Y.Q.); luqiangsdly@163.com (Q.L.); yong-du@csu.edu.cn (Y.D.); 2 Guizhou Aerospace Fenghua Precision Equipment Co., Ltd., Guiyang 550000, China; mingjun_yang@163.com (M.Y.); lhbighead@gmail.com (H.L.); 3 Material Science and Engineering College, Chongqing University of Technology, Chongqing 400050, China; yangmingbo@cqut.edu.cn

**Keywords:** Al-Mg-Si-Cu alloy, rapid quenching, PFZs, grain boundary

## Abstract

Precipitate free zones (PFZs) near grain boundaries generally soften alloys. The quenching rate after solution treatment is an important factor influencing the width of PFZs in Al-Mg-Si-Cu alloy. This study explored the effects of high quenching rates on the grain boundary microstructures and mechanical properties of an Al-Mg-Si-Cu alloy. Samples of various thickness were quenched in water at room temperature and in ethylene glycol at −40 °C, respectively. The results showed that the rapidly quenched samples at −40 °C exhibited better comprehensive mechanical properties than the water-quenched samples. Transmission electron microscopy studies revealed the rapidly quenched samples had wider PFZs, shorter intragranular precipitates, and larger grain boundary precipitates (GBPs) than water-quenched samples. It is proposed that when the quenching rate exceeds the critical cooling rate, e.g., in water quenching or rapid quenching, the formation of PFZs is controlled by the solute depletion mechanism rather than the vacancy depletion mechanism. The nucleation and growth of GBPs thus lead to the depletion of solute atoms, resulting in wider PFZs rather than thinner PFZs according to previous knowledge. This research provides valuable insights into the application of rapid quenching technology for modifying alloys’ microstructures and properties.

## 1. Introduction

Al-Mg-Si-Cu alloy is a key material in lightweight automobile body because of its excellent mechanical properties, machinability, and corrosion resistance [1,2]. Precipitation strengthening is the most important strengthening mechanism in heat-treatable strengthened Al-Mg-Si-Cu alloys [3]. The precipitates formed during the aging process strengthen the alloy by hindering the movement of dislocations. The precipitation sequence of Al-Mg-Si-Cu alloy is the following [4,5]: SSSS → atomic clusters → GP zones → β″, L/S/C, QP, QC → β′, Q′ → Q, Si.

During the process of aging heat treatment, the distribution of precipitates near the grain boundary (GB) in Al-Mg-Si-Cu alloy is non-uniform, forming a special microstructure known as precipitate free zones (PFZs). The formation of PFZs can be explained by the depletion of vacancies near the grain boundary and the depletion of solute atoms caused by the precipitation at the grain boundary [6,7]. PFZs, characterized by the absence of strengthening precipitates, are generally acknowledged as weak regions within the alloy, exhibiting lower strength compared to the grain interior. This inherent property has a considerable influence on the alloy’s mechanical properties and corrosion resistance [8,9].

The composition of grain boundary is closely related to the heat treatment process of the alloy. During the quenching process, quenched-in vacancies will be produced to promote the diffusion of solute atoms. The formation of grain boundary precipitates (GBPs) consumes solute atoms and vacancies, which are both required to form aging precipitates [10,11]. Grain boundary precipitation during quenching is closely related to the cooling rate [10,11]. Previous studies have reported that slow quenching rates can affect the formation of GBPs [12] and also result in an increase in the width of PFZs [11,13]. The quenching rate after solution treatment determines the extent of solute diffusion to the grain boundary and the degree of precipitation at the grain boundary [12]. The quenching rate will not only affect the microstructure parameters, such as the size of intragranular precipitates and grain boundary precipitates, but also affect the mechanical properties of the alloy, such as strength and plasticity.

In the quenching process of Al-Mg-Si-Cu alloy, the grain boundary precipitation consumes a large number of solute atoms, thereby reducing the subsequent precipitation of aging-strengthened precipitates and leading to a decline in the mechanical properties of the alloy [14]. The mechanical properties of the alloy are determined by the supersaturation of the solid solution after quenching. Theoretically, a higher quenching rate is preferred to achieve a higher degree of supersaturation in the solid solution [15]. Quenching at an extremely high cooling rate can retain more solute atoms and vacancies, thus restraining the precipitation of coarse GBPs [16,17]. However, there is a lack of research on the effects of higher quenching rates, beyond water quenching, on the change in grain boundary structure and its impact on the mechanical properties of Al-Mg-Si-Cu alloy. Therefore, it is of great significance to investigate the effects of rapid quenching on the width of PFZs, precipitation behavior, and mechanical properties of Al-Mg-Si-Cu alloy.

In this paper, the microstructure of an Al-Mg-Si-Cu alloy was characterized using transmission electron microscopy (TEM) to study the effect of rapid quenching on the precipitates, PFZs, and mechanical properties of the Al-Mg-Si-Cu alloy. The alloy was quenched in water at room temperature and in ethylene glycol coolant at −40 °C, respectively, and the quenching rate was further increased by reducing the sample thickness. Qualitative microstructural analysis was performed to provide theoretical guidance for process control in the production of the alloy.

## 2. Materials and Methods

The experimental material was a 1.2 mm thick Al-Mg-Si-Cu alloy rolled sheet, and the chemical composition of the alloy was determined using an inductively coupled plasma spectrometer as shown in Table 1.

**Figure 1:**
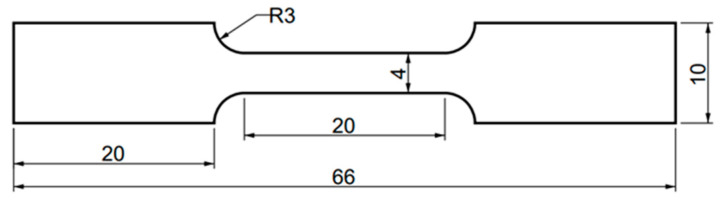
Schematic diagram of the geometric size of tensile samples.

The samples were prepared into plate samples of 10 × 10 mm and 20 × 20 mm and tensile samples of 80 × 10 mm by wire cutting. The geometric shape of the tensile samples is shown in Figure 1. The samples were mechanically ground to thickness of 1 mm, 0.5 mm, and 100 μm.

**Figure 2:**
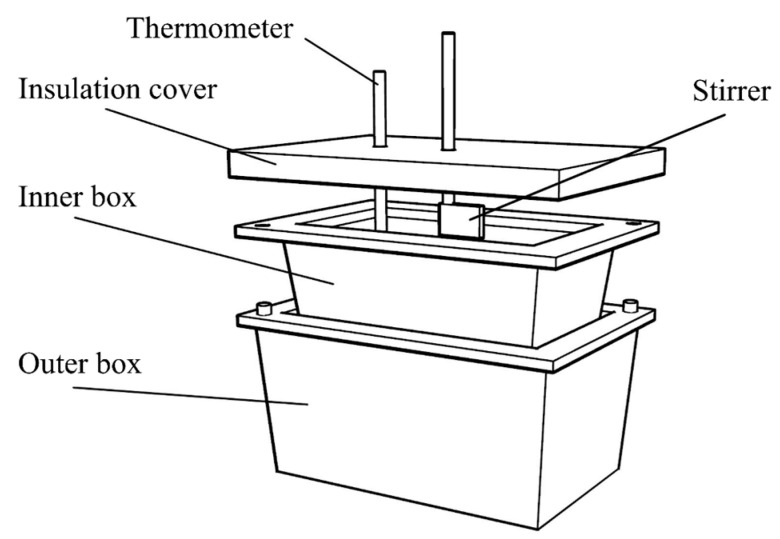
Schematic diagram of the rapid quenching device.

After solid solution heat treatment at 550 °C for 30 min, the samples of varying thickness were quenched in water and ethylene glycol, respectively. Ethylene glycol was placed in the inner box of the quenching apparatus shown in Figure 2, and the samples were quenched after adding liquid nitrogen to the outer box to lower the temperature to −40 °C. Subsequently, the samples were aged at 180 °C. The water-quenched samples with 1 mm thickness and 0.5 mm thickness were designated as 1 mm-WQ and 0.5 mm-WQ, and the rapidly quenched samples with thickness of 1 mm, 0.5 mm, and 100 μm were designated as 1 mm-RQ, 0.5 mm-RQ, and 100 μm-RQ, respectively.

A BUEHLER5104 Vickers hardness tester was used to measure the hardness of the samples. The loading force was 100 g-force, and the loading time was 15 s. Each data point corresponds to the average of six hardness indentations. An Instron 3369 mechanical testing machine was used to perform the uniaxial tensile test at a strain rate of 5 mm/min. Three tensile samples were tested for each group of samples, and the average values were used.

The TEM samples were mechanically thinned and ground to 40~80 μm thickness, and then stamped into 3 mm discs. The samples were twin-jet electropolished in a solution of 30% nitric acid and 70% methanol at −30 °C and 16 V using a Struers TenuPol-5 instrument. FEI Tecnai G2 F20 and Thermo Fisher Talos F200X transmission electron microscopes were used to observe the samples at 160 kV and 200 kV, respectively.

## 3. Results and Discussion

### 3.1. Hardness Evolution during Aging

**Figure 3:**
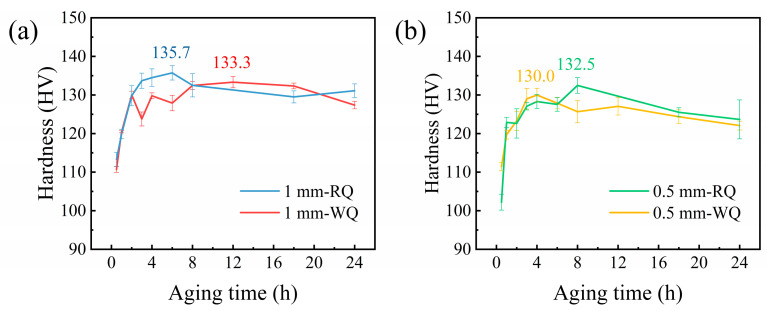
Hardness curves of the Al-Mg-Si-Cu alloy during artificial aging at 180 °C. (**a**) 1 mm samples and (**b**) 0.5 mm samples.

Figure 3 shows the hardness curves of four groups of Al-Mg-Si-Cu alloys artificially aged at 180 °C. It is evident from the figure that the time required to reach the peak-aged state varies depending on the thickness and quenching conditions. The 1 mm-RQ sample achieves peak aging at 6 h with a hardness value of 135.7 ± 1.9 HV, which is the highest among the four groups. The 1 mm-WQ, 0.5 mm-WQ, and 0.5 mm-RQ samples reached peak aging at 12 h, 4 h, and 8 h, respectively, with hardness values of 133.3 ± 1.5 HV, 130.0 ± 1.8 HV, and 132.5 ± 2.0 HV. The 1 mm samples exhibited higher peak-aging hardness than the 0.5 mm samples, and the hardness of 1 mm samples exceeded that of 0.5 mm samples. However, hardness values of samples of different thickness will not be directly compared in this work. Thinner samples should theoretically have better mechanical properties due to faster quenching rates. The mechanical properties of the alloy are determined by the supersaturation of the solid solution after quenching. Theoretically, the higher quenching rate is conducive to achieving a higher degree of supersaturation in the solid solution, which can retain more solute atoms and vacancies, thus inhibiting the precipitation of coarse GBPs [16,17]. This can result in improved mechanical properties by reducing the detrimental effects of grain boundary precipitation on the alloy’s strength and ductility. However, smaller thickness leads to fewer grains in the thickness direction. As a result, due to the uneven distribution of grain sizes, deformation inhomogeneity was increased in thinner samples and negatively impacts the mechanical properties of the alloy [18,19].

Additionally, it was found that the rapidly quenched samples exhibited higher peak-aging hardness compared to the water-quenched samples of the same thickness. This indicates that increasing the quenching rate can improve the hardness of the alloy to some extent. The subsequent studies on the alloy focus on the peak-aging state of the samples.

### 3.2. Tensile Properties

**Figure 4:**
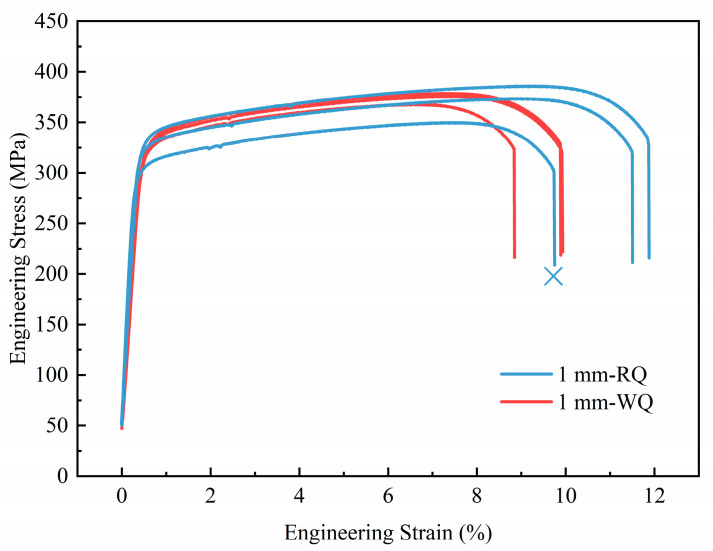
Engineering stress–strain curves of peak-aged samples.

To analyze the impact of quenching rate on the mechanical properties of the Al-Mg-Si-Cu alloy, tensile tests were conducted on two groups of peak-aged samples of 1 mm-WQ and 1 mm-RQ. For each group of samples, we tested three samples. For one of the 1 mm-RQ samples, due to the uncertain factors in the experimental process, there may be defects in the sample and other problems, resulting in a great difference between this sample and the other two samples. We did not include this data (see the cross in Figure 4). Figure 4 shows the engineering stress–strain curves. The ultimate tensile strength, yield strength, and elongation of 1 mm-WQ samples were found to be 374.1 ± 5.6 MPa, 325.5 ± 3.1 MPa, and 9.6 ± 0.6%, respectively. The 1 mm-RQ samples exhibited slightly higher values with an ultimate tensile strength of 379.7 ± 8.8 MPa, yield strength of 326.3 ± 4.0 MPa, and elongation of 11.7 ± 0.3%. Although the differences in yield strength and ultimate tensile strength between the samples are negligible, the 1 mm-RQ samples showed a 2% increase in elongation. Overall, the 1 mm-RQ samples performed better than the 1 mm-WQ samples, indicating an improvement in mechanical properties with rapid quenching.

The critical quenching (cooling) rate for Al-Mg-Si-Cu alloys is widely accepted to be around 10 °C/s [20]. The prevailing belief is that when the quenching rate is higher than the critical cooling rate, a further increase in the quenching rate does not significantly affect the mechanical properties of the aluminum alloy, while a much lower quenching rate leads to inferior mechanical properties [20]. However, Schumacher et al. [21] and Dutta et al. [22] doubt these assumptions and propose that the critical cooling rate is not a linear threshold. They argue for the existence of two distinct boundaries: a lower critical cooling rate, below which nearly all elements in the solid solution precipitate, and a higher critical cooling rate, above which all solute elements remain in a solid solution state.

From the experimental results, both water quenching and rapid quenching exhibit cooling rates higher than the critical cooling rate, resulting in relatively minor changes in mechanical properties. However, it is worth noting that there is still some improvement observed to a certain extent. This aligns with the perspective that when the quenching rate exceeds the critical quenching rate, further increments in the quenching rate will not have a substantial impact on the mechanical properties of the alloy. The difference in elongation will not be discussed here but in the following parts.

### 3.3. Precipitates: Intragranular and at GBs

**Figure 5:**
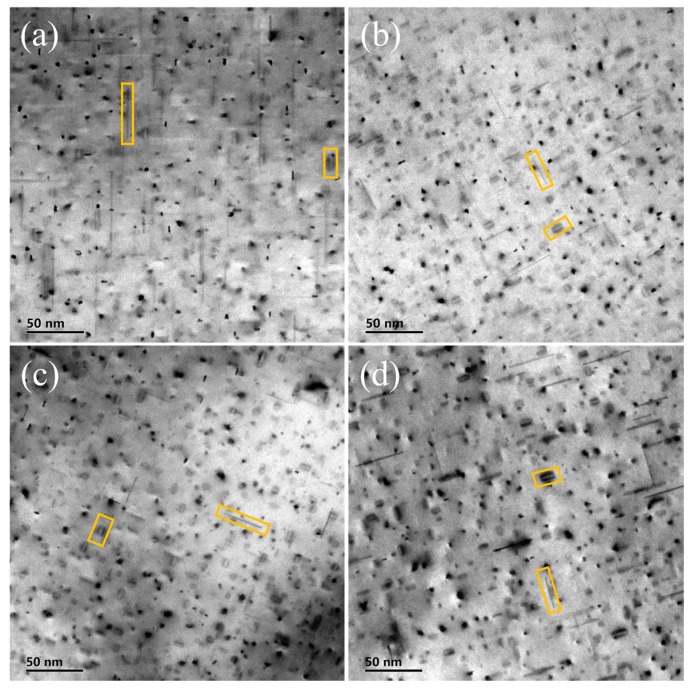
TEM bright-field images of intragranular precipitates in the sample at peak-aged state: (**a**) 1 mm-WQ; (**b**) 1 mm-RQ; (**c**) 0.5 mm-WQ; (**d**) 0.5 mm-RQ. The long rectangular yellow box indicates the late-like precipitates, and the short rectangular yellow box indicates needle-like precipitates.

The TEM bright-field images of the intragranular precipitates in the peak-aged state of different samples are shown in Figure 5, all observed along the <100>_Al_ direction. According to the aging precipitation sequence and the bright-field image characteristics of precipitates in Al-Mg-Si-Cu alloys, there are three types of precipitates, i.e., β″ phase, Q′ phase, and L/C phase, in the peak-aged state of the alloy [23]. The β” phase is needle-like and serves as the primary strengthening precipitate of Al-Mg-Si-Cu alloys, which can be identified by its circular end-on view and the surrounding strain contrast when viewed side-on in TEM [24]. The Q′ phase and L/C phase are lath-like, which can be recognized by their rectangular end-on view with different habit planes [23,25]. The late-like precipitates are highlighted within the long yellow rectangular boxes in Figure 5, while the short rectangular box indicates needle-like precipitates.

**Figure 6:**
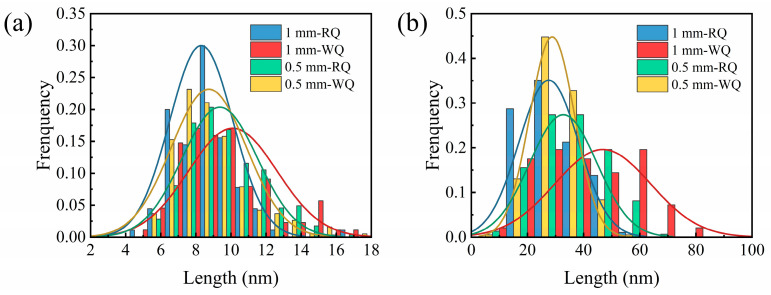
The length distribution frequency of intragranular precipitates in the sample at peak-aged state: (**a**) needle-like; (**b**) lath-like.

The lengths of lath-like and needle-like precipitates were measured for four groups of samples, respectively. The length distribution of the precipitates was shown in Figure 6. The average length of short needle-like precipitates in the 1 mm-WQ, 1 mm-RQ, 0.5 mm-WQ, and 0.5 mm-RQ samples were 10.1 ± 2.5 nm, 8.3 ± 1.8 nm, 8.7 ± 2.1 nm, and 9.4 ± 2.2 nm, respectively. Furthermore, the average length of the long lath-like precipitates was 46.8 ± 17.1 nm, 27.6 ± 10.6 nm, 28.8 ± 8.0 nm, and 32.8 ± 12.0 nm, respectively. The length of precipitates in 1 mm-WQ sample is greater than that of other three groups of samples, regardless of whether they are needle-like or lath-like. These results indicate that the precipitates can be refined with an increase in quenching rate. Refinement of precipitates always produces an increase in their number density since the total amount of solutes for precipitate formation is limited. Qualitatively speaking, the number density of precipitates in the 1 mm-WQ sample is indeed lower than in other samples, according to the comparison between Figure 5a,b–d.

The precipitation-strengthening mechanism of Al-Mg-Si-Cu alloys has been extensively investigated, with the peak strength primarily attributed to the formation of the β″ phase. Quenching after solution treatment produces quenched-in vacancies, which promotes the diffusion of solute elements into clusters and their growth into metastable precipitates during the subsequent aging process, and the concentration of vacancies determines the precipitation-strengthening behavior of the alloy [26,27]. Generally, the slower quenching rate will lead to lower strength because the slow quenching rate produces fewer quenched-in vacancies, and the quenched-in vacancies can promote the diffusion of solute atoms. Due to the lack of quenched-in vacancies, the nucleation rate of precipitates is lower, and the formation of precipitates may be delayed [11,28]. Additionally, a slow quenching rate can result in the precipitation of coarse second phase particles and GBPs, and the formation of these phases consumes part of the solute, resulting in less solute that can be used for intragranular strengthening precipitates and lower numerical density of these precipitates, resulting in lower strength after peak aging [29,30].

When the quenching rate is above 10 °C/s, it has little effect on the density of the nano-precipitates in the microstructure of Al-Mg-Si-Cu alloy [20]. The water-quenched sample roughly underwent a cooling rate of 250 °C/s [13,31]. In this study, among the rapidly quenched and water-quenched samples, of which the quenching rates are apparently higher than 10 °C/s, the intragranular precipitates of the rapidly quenched sample are shorter than that of the water-quenched sample, and the density is also different to some extent.

**Figure 7:**
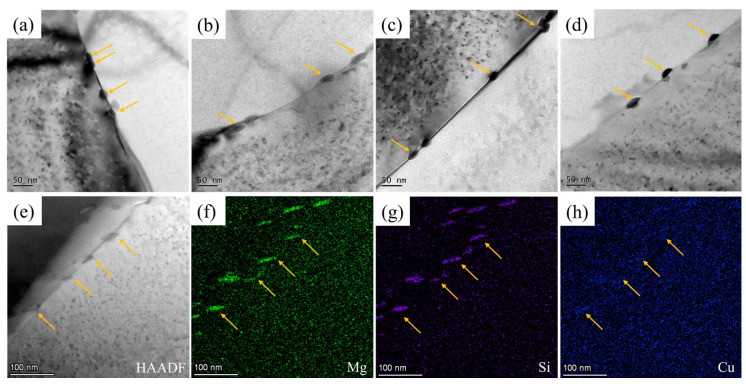
TEM bright-field images of grain boundary precipitates in peak-aged samples: (**a**) 1 mm-WQ; (**b**) 1 mm-RQ; (**c**) 0.5 mm-WQ; (**d**) 0.5 mm-RQ; and HAADF-STEM and EDX mapping of 100 μm-RQ peak-aged samples (**e**–**h**). The yellow arrows indicate grain boundary precipitates.

Furthermore, a small number of GBPs with a length of tens of nanometers were observed in all four groups of samples. Figure 7 shows the bright-field images of these GBPs and high angle annular dark field-scanning transmission electron microscopy (HAADF-STEM) images and energy-dispersive X-ray spectroscopy (EDX) elemental maps of GBPs in the 100 μm-RQ sample (aged for 6 h at 180 °C, similar to 1 mm-RQ). The GBPs are rich in Si, Mg, and Cu elements. The phase is Q’/Q phase that contains much more Mg and Si than Cu [32], according to the recently reported composition of Al_6_Mg_6_Si_7.2_Cu_2_ [33]. The GBPs are places of nucleation, growth, and aggregation of damage, which are detrimental to the mechanical properties. The equivalent diameters of GBPs of 1 mm-WQ, 1 mm-RQ, 0.5 mm-WQ, 0.5 mm-RQ, and 100 μm-RQ samples at peak-aged state were 17.8 ± 1.6 nm, 22.9 ± 4.9 nm, 21.7 ± 3.6 nm, 19.8 ± 3.4 nm, and 19.4 ± 0.8 nm, respectively. The 1 mm-WQ sample exhibited the smallest size of GBPs.

The formation mechanism of GBPs involves the depletion of solutes around grain boundaries. Grain boundaries serve as heterogeneous nucleation points and rapid diffusion channels for precipitates. Heterogeneous precipitation is favored on grain boundaries [34,35]. The solute moves from the surrounding matrix to the grain boundary and then diffused along the grain boundary, resulting in the growth/coarsening of GBPs. In this study, the 1 mm-WQ sample, with the slowest cooling rate, had the smallest GBPs, while the rapidly quenched samples exhibited coarser GBPs. This result can be explained from two aspects. Firstly, the rapidly quenched samples had a higher level of supersaturated solid solubility, resulting in a greater driving force for precipitation. This increased driving force led to the formation of larger precipitates at the grain boundaries. Secondly, the rapid quenching process introduced a higher concentration of vacancies in the material. These vacancies acted as facilitators for the diffusion of solute atoms. As a result, solute atoms were able to reach the grain boundaries more quickly, promoting the formation of larger GBPs.

### 3.4. Precipitate Free Zones

**Figure 8:**
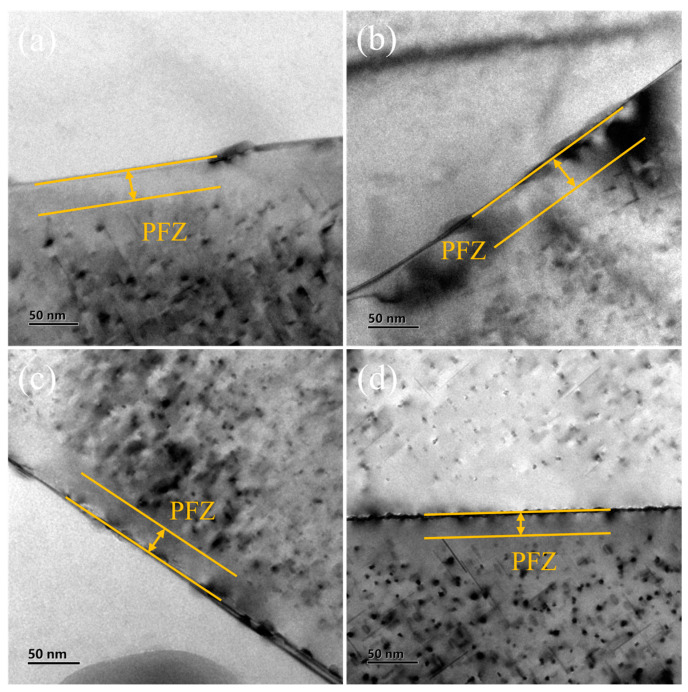
TEM bright-field images of PFZs in the peak-aged state: (**a**) 1 mm-WQ; (**b**) 1 mm-RQ; (**c**) 0.5 mm-WQ; (**d**) 0.5 mm-RQ.

Figure 8a–d shows the typical TEM bright-field images of PFZs in the peak-aged state of 1 mm-WQ, 1 mm-RQ, 0.5 mm-WQ, and 0.5 mm-RQ samples. Due to the different orientations of the two grains near the grain boundary, it is challenging to adjust the two grains to the <001>_Al_ axis at the same time, and only in some grains can the precipitates be observed in the two grains at the same time. Therefore, this study considers the certain symmetry of PFZs at both sides of a GB and only measures the width of PFZs on one side. Different grain boundaries are measured at least eight times in each sample.

The average widths of PFZs in 1 mm-WQ, 1 mm-RQ, 0.5 mm-WQ, and 0.5 mm-RQ samples were 30.4 ± 4.4 nm, 34.3 ± 6.0 nm, 34.5 ± 4.1 nm, and 33.3 ± 7.9 nm, respectively. Interestingly, the PFZs width of the 1 mm-WQ sample is smaller than those in the other three samples, indicating that increasing the quenching rate beyond water-quenching rate resulted in wider PFZs.

There has been disagreement about the formation mechanism of PFZs, and there are two main mechanisms: vacancy depletion mechanism and solute depletion mechanism. The solute depletion mechanism suggests that during aging, solute atoms migrate toward grain boundary and form GBPs, resulting in the depletion of solute atoms in local regions and subsequent formation of PFZs [36,37,38]. The vacancy depletion mechanism suggests that vacancies facilitate the heterogeneous nucleation of precipitates, and the formation of GBPs consumes vacancies near grain boundary, resulting in a decrease in the concentration of vacancies and the inability of precipitates to nucleate at grain boundary vicinities, thus leading to the formation of PFZs. 

Contrary to the general understanding, it is found in this work that the rapid quenching rate resulted in wider PFZs instead of narrowing them, and meanwhile, the mechanical properties of the alloy were slightly improved. This may be related to the formation of GBPs. The 1 mm-WQ samples had the smallest size of GBPs and the narrowest PFZs. The vacancy depletion mechanism suggests that the nucleation of GBPs is influenced by vacancy concentration. However, both rapid quenching and water quenching can generate a high density of quenched-in vacancies. According to the study of Liu et al. [39], after quenching, vacancy-related defects, such as vacancies or vacancy-associated solute atoms/clusters, account for more than 80% of the positron annihilation signal. This indicates that these vacancy-related defects are well-preserved during the quenching process and the vacancy is not poor. Furthermore, the study also suggests that after 80 min of natural aging, all possible solute clusters have formed, regardless of the quenching rate, indicating that vacancies are not depleted even after aging. Therefore, the solute atom depletion mechanism is considered to explain the experimental phenomenon observed in this work. Rapid quenching produces more quenched-in vacancies than water quenching. These vacancies can facilitate the diffusion of solute atoms towards the grain boundary to form GBPs, and the GBPs preferentially nucleate and grow at the GBs, resulting in a depletion of solute atoms near the grain boundary. As a result, wider PFZs and larger GBPs were formed, and meanwhile, finer and denser intragranular precipitates were grown. The latter accounts for the slight increase in strength and hardness, while the wider PFZs explain the improvement in plasticity since dislocations slide across wider PFZs more easily.

## 4. Conclusions

In this study, the effects of rapid quenching at −40 °C on the microstructure and mechanical properties of an Al-Mg-Si-Cu alloy were investigated. Especially, intragranular precipitates, GBPs, and PFZs were qualitatively analyzed using TEM. The results are summarized as follows:

(1) The mechanical properties, including hardness, strength, and plasticity of rapidly quenched samples are better than those of water-quenched samples. It is shown that even when the quenching rate exceeds the critical cooling rate of 10 °C/s suggested in literature, increasing the quenching rate can improve the mechanical properties of the alloy. However, the increase in hardness and strength is negligible.
(2) When the quenching rate exceeds the critical cooling rate, the PFZs in the peak-aged state of rapidly quenched samples were observed to be wider compared to those in the water-quenched samples. Additionally, the size of GBPs was found to be larger, while the size of intragranular precipitates was smaller.
(3) When the quenching rate exceeds the critical cooling rate, it is speculated that the formation of PFZs is controlled by the solute depletion mechanism rather than the vacancy depletion mechanism. The nucleation and growth of GBPs lead to the depletion of solute atoms, resulting in wider PFZs at a higher quenching rate.
(4) A finer and denser distribution of intragranular precipitates accounts for the slight increase in strength and hardness mentioned above, while the wider PFZs explain the notable increase in plasticity.

## Figures and Tables

**Table 1:** Chemical composition of the investigated samples (wt. %).

Element	Mg	Si	Cu	Mn	Fe	Al
Measured Composition	1.12	0.78	0.56	0.51	0.47	Bal.

## Data Availability

Data will be made available on request.
